# Long-term outcome and patterns of failure in patients with advanced head and neck cancer

**DOI:** 10.1186/1748-717X-6-70

**Published:** 2011-06-10

**Authors:** Henrik Hauswald, Christian Simon, Simone Hecht, Juergen Debus, Katja Lindel

**Affiliations:** 1 Department of Radiation Oncology, University of Heidelberg, Heidelberg, Germany; 2 Department of Oto-Rhino-Laryngology, University of Heidelberg, Heidelberg, Germany

**Keywords:** HNSCC, head and neck cancer, radiotherapy, radiochemotherapy, irradiation, long-term follow-up

## Abstract

**Purpose:**

To access the long-time outcome and patterns of failure in patients with advanced head and neck squamous cell carcinoma (HNSCC).

**Methods and materials:**

Between 1992 and 2005 127 patients (median age 55 years, UICC stage III n = 6, stage IV n = 121) with primarily inoperable, advanced HNSCC were treated with definite platinum-based radiochemotherapy (median dose 66.4 Gy). Analysed end-points were overall survival (OS), disease-free survival (DFS), loco-regional progression-free survival (LPFS), development of distant metastases (DM), prognostic factors and causes of death.

**Results:**

The mean follow-up time was 34 months (range, 3-156 months), the 3-, 5- and 10-year OS rates were 39%, 28% and 14%, respectively. The median OS was 23 months. Forty-seven patients achieved a complete remission and 78 patients a partial remission. The median LPFS was 17 months, the 3-, 5- and 10-year LPFS rates were 41%, 33% and 30%, respectively. The LPFS was dependent on the nodal stage (p = 0.029). The median DFS was 11 months (range, 2-156 months), the 3-, 5- and 10-year DFS rates were 30%, 24% and 22%, respectively. Prognostic factors in univariate analyses were alcohol abuse (n = 102, p = 0.015), complete remission (n = 47, p < 0.001), local recurrence (n = 71, p < 0.001), development of DM (n = 45, p < 0.001; median OS 16 months) and borderline significance in nodal stage N2 versus N3 (p = 0.06). Median OS was 26 months with lung metastases (n = 17). Nodal stage was a predictive factor for the development of DM (p = 0.025). Cause of death was most commonly tumor progression.

**Conclusions:**

In stage IV HNSCC long-term survival is rare and DM is a significant predictor for mortality. If patients developed DM, lung metastases had the most favourable prognosis, so intensified palliative treatment might be justified in DM limited to the lungs.

## Introduction

The incidence of oropharyngeal cancer in German men in 2004 was 16.3 per 100.000 [1]. Smoking and alcohol consumption were known risk factors for the development of head and neck squamous cell carcinoma (HNSCC)[2,3]. New and optimized treatment methods increase loco-regional progression-free survival (LPFS) and disease-free survival (DFS) in patients with advanced head and neck carcinomas and thereby overall survival (OS) in the short-term follow-up [4-7]. Data on long-term follow-up and patterns of failure are rare [8]. The published incidence of distant metastases (DM) in HNSCC is widespread and varies between 6% and 47%[9-14]. Spector et al published e. g. an incidence of 8.5% in 2550 patients treated for squamous cell carcinomas of the larynx and hypopharynx between 1971 and 1991 [14]. The published incidence of DM in a subgroup of patients with stage IV disease was even as high as 55%[15]. Reported factors influencing the incidence of DM were tumor stage, especially the extension of nodal disease, histological patterns and loco-regional tumor control [9,16-18]. Lim et al reported that the presence of pathologic lymph nodes, especially bilateral neck metastases, was an independent risk factor for the development of DM in oral and oropharyngeal squamous cell carcinomas [16]. The leading site for DM were the lungs, followed by the skeletal system [9,14]. So DM might become a relevant problem and data on outcome is warranted to improve the adaption of the treatment. This retrospective study performs uni- and multivariate analyses on the outcome of patients treated with concurrent platinum-based, hyperfractionated-accelerated radiochemotherapy for primarily inoperable, advanced HNSCC according to the treatment protocol of Staar et al. [19]. Furthermore factors possibly impacting on the development of DM in patients with advanced HNSCC were analyzed to identify subgroups, in which additional diagnostic and/or therapeutically options might improve prognosis, morbidity and mortality.

## Patients and methods

### Patient characteristics

From 1992 to 2005 127 patients (median age 55 years, range 32-79 years; male n = 110, female n = 17) were treated according to the treatment protocol of Staar et al. [19] with a definite platinum-based concurrent hyperfractionated-accelerated radiochemotherapy for primarily inoperable, advanced oro- (n = 41) and hypopharyngeal (n = 86) squamous cell carcinoma at the Department of Radiation Oncology of the University Hospital Heidelberg. Patients treated with other treatment regimes for the same disease were excluded. All patients were initially staged as free of DM. Further patient characteristics are listed in table 1.

**Table 1:** Patient characteristics

Patient characteristic	No. of patients	Percentage
**Gender**		
Male	110	87
Female	17	13
**Tumor localization**		
Oropharyngeal	86	68
Hypopharyngeal	41	32
**Etiologic factors**		
Alcohol abuse	102	80
Tobacco abuse	99	78
**HPV16/p16**		
Positive	5	12
Negative	38	88
**TNM-Staging**		
T2	9	7
T3	24	19
T4	94	74
Tx	3	2
N0	7	6
N1	6	5
N2 (a/b/c)	97 (2/35/60)	77 (2/28/47)
N3	17	13
Tumor stage according to UICC classification 1997		
III IVA	6 104	5 82
IVB	17	13

### Diagnostic work-up and Treatment

The initial workup included physical and laboratory examinations, imaging procedures, such as x-ray studies, ultrasound (US), magnetic resonance imaging (MRI) or computerized tomography scans (CT) as well as biopsies. Positron-emission tomography (PET) was not performed on a regular base. Data on HPV16/p16 was retrospectively accessible in 43 (34%) of the patients. Five of these patients were HPV16/p16 positive. The treatment consisted of a concurrent hyperfractionated-accelerated radiotherapy and platinum-based chemotherapy. Irradiation was planned using two- or three-dimensional-based techniques and controlled by simulator-based imaging. Patient immobilization was done by thermoplastic masks. Megavolt radiotherapy was administered by linear accelerators to a median dose of 66.4 Gy (range, 59.4-70.3 Gy). The median time interval between initial diagnosis and first irradiation was 25 days. Chemotherapy consisted of 5-FU (600 mg/m^2 ^body surface) as a continuous infusion and carboplatinum (70 mg/m^2 ^body surface) as short-term infusion day 1-5 and 29-33. Ten patients had to quit chemotherapy early due to toxicity (n = 2), personal wish (n = 2) or undocumented reasons (n = 6). Regular follow-up examinations included clinical examination, US, MRI or CT and were classified as complete remission (CR, requiring no detectable disease), partial remission (PR, tumor mass reduction of at least 50%), no response (NR, less than 50% tumor mass reduction) or as progressive disease (PD). The first follow-up examination was scheduled 6 to 8 weeks after radiotherapy was finished. Radiooncological treatment time ranged between 31 and 80 days (median 40 days).

### Statistics

The tumor was staged according to the TNM classification recommended by the International Union against Cancer (UICC) 1997. The latter was analysed regarding overall survival (OS), disease-free survival (DFS), loco-regional progression-free survival (LPFS), distant metastases-free survival (DMFS) and causes of death. Statistical analyses were carried out with SPSS statistical package (SPSS Inc., Chicago, IL, U.S.A.) using log-rank test (Mantel-Cox), Kaplan-Meier's estimation, multivariate Cox-regression analysis (backwards stepwise, p out >0.1, factors included: total dose of irradiation (>/= or < 66,4Gy); treatment time (>/= or <40 days); alcohol abuse; tobacco abuse; age (>/= or <55 years); Stage IVa versus IVb; stage N2 versus N3; localization oro- versus hypopharynx; CR versus PR; distant metastases; loco-regional recurrence) and Fisher's exact test. Significance was defined as p-value < 0.05. All time estimates began with the initiation of radiation treatment. Documented long-term side effects were classified according to the RTOG/EORTC Late Radiation Morbidity Scoring Scheme (Appendix IV, CTC Version 2.0).

## Results

### Response to treatment and loco-regional control

The mean follow-up time was 34 months (range, 3-156 months). Forty-seven patients (37%; n = 29 hypopharyngeal- and n = 18 oropharyngeal carcinoma) achieved a complete remission, whereas 78 patients (61%; n = 55 hypopharyngeal- and n = 23 oropharyngeal carcinoma) showed a partial remission. One patient (1%) had progressive disease. No treatment response was available in one patient (1%). The median LPFS was 17 months, the 3-, 5- and 10-year LPFS rates were 41%, 33% and 30%, respectively. The median LPFS was significantly different (p = 0.029) in patients with N0 disease (20 months), N1 disease (43 months), N2 disease (18 months) and N3 disease (7 months).

### Distant metastases and distant metastases-free survival

**Figure 1:**
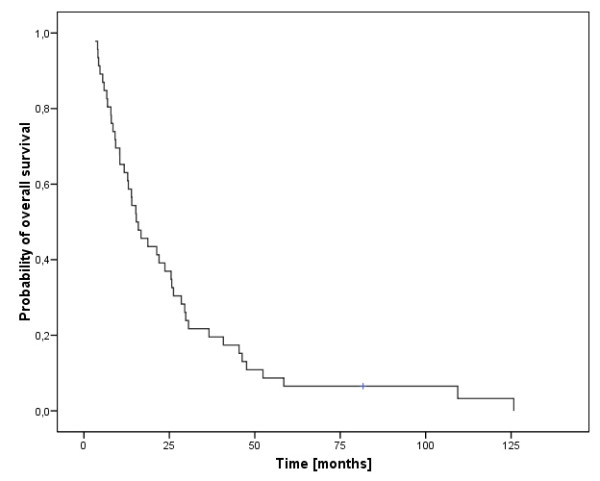
**Overall survival of 45 patients with development of distant metastases**.

**Figure 2:**
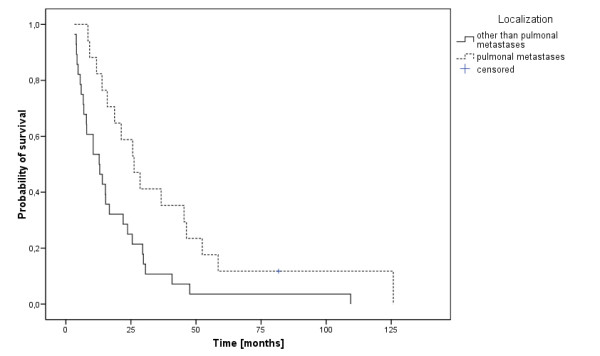
**Survival of patients with pulmonal (n=17) versus elsewhere located (n=28) metastases**.

Distant metastases-free survival was median 66 months (range, 2-156 months). Forty-five of our patients (35%; 41 male and 4 female; mean age 55 years, range 37-79 years) were diagnosed with distant metastases in the median 8 months after initial diagnosis. The nodal stage in these 45 patients was distributed as follows: N0 n = 4, N1 n = 0, N2a/b n = 17, N2c n = 17, N3 n = 8. Diagnosis of DM was primarily based on imaging procedures, such as x-ray studies and CT scans. The locations of DM were most commonly the lungs (38%), followed by multiple locations (36%), the skeletal system (11%), liver (9%), brain (4%) and skin (2%). Palliative treatment regimes most commonly included different systemic therapies, in localized DM additionally palliative irradiation or stereotactic radiotherapy but also surgical procedures like metastasectomy. The development of DM led to a significantly shorter median OS time compared to 38 months without DM (p < 0.001). The median OS in the 45 patients with DM was 15.6 months (figure 1, range 3-126 months) and the one year-overall survival rate 72%. Patients with lung metastases had a median OS of 26 months, compared to 14 months in patients with multiple locations, 13 months with metastases to the skeletal system, 21 months with liver metastases, 7 months with brain metastases and 15 months with skin metastases. There was a significant one-year-survival difference between patients with lung metastases (82%) and other metastatic locations (brain 0%, multiple locations 56%, liver 50% and bone 60%, p = 0.01, log rank, figure 2). There was no difference in OS for patients with DM from oro- or hypopharyngeal cancer (p = 0.51). The stage of nodal disease had significant influence on OS (the median OS in N0-stage was 13 months, compared to 30 months in N2a/b-stage and 8 months in N3-stage, p = 0.025). We did not find a significant prognostic factors for the development of DM regarding gender (p = 0.29, Fisher's exact test), age (p = 0.85, Fisher's exact test), tumor localization (p = 0.89, Fisher's exact test) and treatment response (p = 0.23, Fisher's exact test). Chronic alcohol (tobacco) abuse was not accessible in this subgroup due to the fact that 44 (40) of the 45 patients showed chronic alcohol (tobacco) abuse. Local recurrence occurred in 28 patients (62%) in addition to their DM. There was no significant difference regarding OS of patients with DM alone compared to patients with LR and DM (1-year survival 53% and 58%, respectively).

### Survival

**Figure 3:**
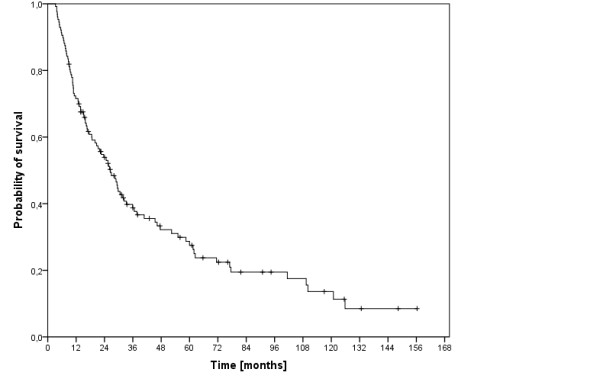
**Overall survival of 127 patients with primarily inoperable, advanced HNSCC**.

At last follow-up, 33 patients (26%) were still alive and 94 patients (74%) had passed. The median overall (disease free) survival time was 27 months (11 months) and the 3-, 5- and 10-year overall (disease free) survival rates were 39% (30%), 28% (24%) and 14% (22%), respectively (figure 3). The cause of death was tumor dependent in 69 patients (73%). In 4 patients (4%) the cause of death was another carcinoma and in one patient each (1%) cardiac insufficiency and pulmonary embolism. In 19 patients (20%) the cause of death was not documented.

**Figure 4:**
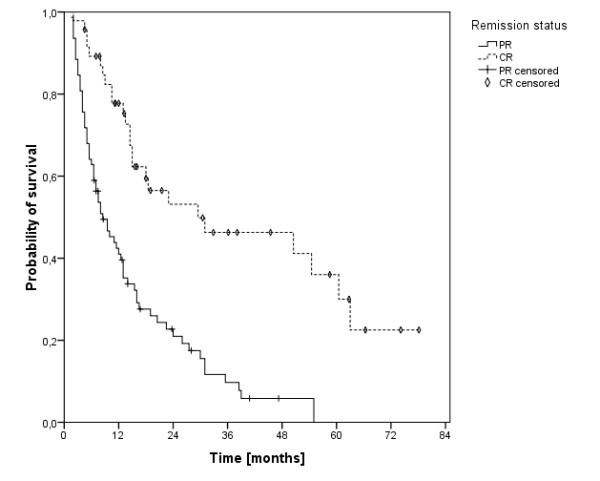
**Survival of patients with a complete remission (n=47) versus partial remission (n=78)**.

The univariate analysis on the influence of UICC tumor stage on OS showed a borderline significance for patients with stage IVA disease versus IVB (p = 0.06). OS in patients with N2 disease (median 29 months, 3-, 5- and 10-year-OS was 42%, 28% and 15%, respectively) a borderline significantly longer OS compared to patients with N3 disease (median 11 months, 3-, 5- and 10-year-OS was 29%, 22% and 11%, respectively; p = 0.06). The localization of the primary tumor, whether hypo- or oropharyngeal, had no significant influence on the OS (median 26 vs. 29 months, p = 0.55). One other univariate prognostic factor was alcohol abuse (n = 102, p = 0.015). Further more, patients with a CR had a significantly improved OS compared to patients with a PR (median 59 months versus 17 months, p < 0.001, figure 4). We did not find a significant influence on OS by tobacco abuse (p = 0.44), age >/= 55 years (p = 0.45), median treatment dose >/= 66.4 Gy (p = 0.5) and total radiooncological treatment time >/= 40 days (p = 0.7). The sample of patients who were HP16/p16 positive was too small for useful statistical analysis. The results of the uni- and multivariate analyses were shown in table 2 and table 3, respectively.

**Table 2:** Results of the univariate analyses

Factor	p-value
Stage IVa versus IVb	0.06
Stage N2 versus N3	0.06
Total dose of irradiation (>/= or <66,4Gy)	>0.1
Total radiooncological treatment time (>/= or <40 days)	0.7
Complete versus partial remission	<0.001
Age (>/= or <55 years)	>0.1
Alcohol abuse	0.015
Secondary primary tumors	>0.1

**Table 3:** Results of the multivariate analyses on LPFS, DFS and OS

Factor	p-value LPFS	p-value DFS	p-value OS
Stage IVa versus IVb	>0.1	>0.1	0.16
Stage N2 versus N3	0.045	>0.1	>0.1
CR versus PR	<0.001	<0.001	<0.001
Distant metastases	>0.1	--	0.01
Loco-regional recurrence	--	--	0.006
Age (>/= or <55 years)	0.041	0.003	>0.1
Alcohol abuse	>0.1	0.027	>0.1

### Long-term side effects

Most common long-term side effects documented were xerostomia and alterations in taste. At last follow-up, 17 of the 33 patients who were still alive (51%) reported grade III to IV xerostomia.

### Second primary carcinoma

Second primary carcinomas developed in 27 patients (21%). Their most common location was the head and neck region (n = 9), followed by the esophagus (n = 6), lungs (n = 5) and stomach (n = 2). One patient each developed a hepatocellular-, pancreatic-, penile-, prostatic- and renal cell carcinoma. Patients with secondary carcinomas did not have a significantly longer survival than those without secondary tumors (46 months versus 25 months, p = 0.26).

## Discussion

We report on a retrospective analysis of the treatment results in 127 patients treated with concurrent, platinum-based, hyperfractionated-accelerated radiochemotherapy between 1992 and 2005 for primarily inoperable advanced oro-and hypopharyngeal squamous cell carcinoma. A treatment regime for locally advanced oro- and hypopharyngeal squamous cell carcinoma is a definite concurrent platinum-based radiochemotherapy. In the daily routine, guidelines regarding the optimal treatment of the patients, including those with DM, are warranted. This study's aim was to evaluate the long-term treatment outcome at our institution as well as patterns of failure and help finding ways to improve prognosis, morbidity and mortality in patients with advanced HNSCC.

The treatment regime used in our patients was based on the prospective and multicentre trial on radiotherapy in advanced head and neck cancer initially published by Staar et al [19]. After accelerated and hyperfractionated radiotherapy with concurrent 5-FU and carboplatinum chemotherapy the authors achieved a 1- and 2-year OS rate of 66% and 48%, respectively. The total response to treatment was above 90%. The rate of xerostomia 1 year after treatment was 66%. An update on the report was recently published by Semrau et al [20]. The reported 5-year overall survival rate was 25.6% and the median survival 23 months. In a trial on concomitant radiochemotherapy in advanced oropharyngeal cancer Denis et al reported an median survival of 20 months and a 5-year overall survival rate of 22% for patients treated with concomitant radiochemotherapy [21]. The 3-, 5- and 10-year OS rates of 39%, 28% and 14%, respectively as well as the median OS of 23 months in our cohort were comparable and in good agreement to the published data.

Adelstein et al reported on 222 patients with advanced head and neck squamous cell carcinoma treated with a multiagent concurrent radiochemotherapy with 5-FU and cisplatin during weeks 1 and 4 [22]. The tumor was located in the oropharynx in 52%. The 5-year OS rate was 65%. This superiority of the results by Adelstein et al may be due to the selection, since preserving organ function was one mayor concern and patients with tumor-invasion into the bone or cartilage were not considered appropriate for this treatment approach. In their report on 81 patients treated with hypofractionated accelerated radiotherapy and concurrent chemotherapy for advanced HNSCC (including larynx, oral cavity, oro- and hypopharynx) Sanghera et al reported a 2-year OS rate of 67.8% in 68 patients with UICC stage III and IV [23]. The superiority of these results may be due to the lower count of T4 tumors (25/81 patients) and lower count of N2c or N3 disease (14/81 patients) in the cohort of Sanghera et al. Improvements in survival with 1- and 2-year OS rates of up to 81.5% and 71.6% and loco-regional tumor relapse rates of 33-35% were found in studies on concomitant boost accelerated radiation regimes with concomitant cisplatin [4,8].

As seen in our results as well as in earlier reports, there is a high incidence of persistent xerostomia which could negatively influence quality of life. An actual approach of reducing side effects of radiation therapy was published by Teguh et al. [24]. The authors concluded that hyperbaric oxygen therapy shortly after finishing radiation therapy is an effective option for reducing radiation-induced side effects.

In the question of factors influencing the incidence of DM different variables as tumor stage, histological patterns and loco-regional tumor control were reported. Best predictor for overall survival and distant failure as reported by Brockstein et al was the stage of nodal disease [25]. Leon et al analysed 1244 patients with loco-regionally controlled head and neck cancers. They found N-stage, T-stage and the localization of the tumor at hypopharynx or supraglottis to be variables increasing the incidence of DM on multivariate analysis [26]. In the multivariate analysis of Lim et al the presence of pathologic positive lymph node, especially bilateral neck metastases, was an independent risk factor for the appearance of isolated distant metastases in oral and oropharyngeal squamous cell carcinoma [16]. In our patient group, the stage of nodal disease was a significant predictor for survival (p = 0.025), but neither primary tumor localization (p = 0.89), nor treatment response (p = 0.23) or age (p = 0.85) were significantly related to the development of distant metastases. This finding might be due to the fact of a relatively small cohort. Extracapsular tumor spread and histological grading were retrospectively not accessible.

The most common site of distant metastases in previously published data as well as found in our cohort's findings were the lungs [9,14,27]. Furthermore, in the report by Alvi et al. DM developed after a mean time of 15 months and survival was 5 months after diagnosis of DM [27]. Median time to distant failure (median 8 months) and median OS (median 16 months) in our cohort were comparable, keeping in mind that the time estimation in our analysis started at the initial diagnosis of the oro- or hypopharyngeal carcinoma. In general the salvage rates for distant failure were poor. Spector et al reported a curing rate of 16% in pyriform carcinoma with early solitary focal DM [14]. A 5-year survival rate of 43% after surgical resection achieved Mazer et al on 44 patients with pulmonary metastases from upper aerodigestative tract cancer [28]. Finley et al reported on their evaluation of surgical resection of pulmonary metastases of head and neck cancer that a resection of a solitary pulmonary metastasis resulted in long-term survival in selected patients [29]. Since treatment after diagnosis of DM was palliative and individual in most cases in our cohort, it was not useful to analyze the different treatment approaches in the situation of DM.

## Conclusion

Hyperfractionated-accelerated radiotherapy with concurrent platinum-based chemotherapy is an effective treatment option and offers a chance for long-term survival for patients with primarily inoperable, advanced HNSCC, which is still rare. New and optimized treatment methods increase loco-regional tumor control in patients with advanced head and neck carcinomas and thereby survival. So stage IV patients might be diagnosed with DM and this might become a relevant problem in achieving long-term control. Patients with DM restricted to the lungs had the most favourable prognosis compared to patients with other metastatic locations. Intensified palliative treatment might be justified especially in cases of DM limited to the lungs.

## Competing interests

The authors declare that they have no competing interests.

## Authors' contributions

HH: analysis and interpretation of data, writing manuscript. CS: critically revision for important intellectual content, interpretation of data. Simone Hecht: acquisition and analysis of data. JD: critically revision for important intellectual content, interpretation of data. KL: substantial contributions to conception and design; critically revision for important intellectual content; final approval for publication. All authors have read and approved the final manuscript.
